# Determinants of tick-borne encephalitis in counties of southern Germany, 2001-2008

**DOI:** 10.1186/1476-072X-9-42

**Published:** 2010-08-13

**Authors:** Christian Kiffner, Walter Zucchini, Philipp Schomaker, Torsten Vor, Peter Hagedorn, Matthias Niedrig, Ferdinand Rühe

**Affiliations:** 1 Department of Forest Zoology and Forest Conservation incl. Wildlife Biology and Game Management, Büsgen-Institute, Georg-August-University Göttingen, Büsgenweg 3, 37077 Göttingen, Germany; 2 Institute for Statistics and Econometrics, Georg-August-University Göttingen, Platz der Göttinger Sieben 5, 37073 Göttingen, Germany; 3 Centre for Biological Safety, Robert Koch-Institut, Nordufer 20, 13353 Berlin, Germany

## Abstract

**Background:**

Tick-borne encephalitis (TBE) virus can cause severe symptoms in humans. The incidence of this vector-borne pathogen in humans is characterised by spatial and temporal heterogeneity. To explain the variation in reported human TBE cases per county in southern Germany, we designed a time-lagged, spatially-explicit model that incorporates ecological, environmental, and climatic factors.

**Results:**

We fitted a logistic regression model to the annual counts of reported human TBE cases in each of 140 counties over an eight year period. The model controlled for spatial autocorrelation and unexplained temporal variation. The occurrence of human TBE was found to be positively correlated with the proportions of broad-leafed, mixed and coniferous forest cover. An index of forest fragmentation was negatively correlated with TBE incidence, suggesting that infection risk is higher in fragmented landscapes. The results contradict previous evidence regarding the relevance of a specific spring-time temperature regime for TBE epidemiology. Hunting bag data of roe deer (*Capreolus capreolus*) in the previous year was positively correlated with human TBE incidence, and hunting bag density of red fox (*Vulpes vulpes*) and red deer (*Cervus elaphus*) in the previous year were negatively correlated with human TBE incidence.

**Conclusions:**

Our approach suggests that a combination of landscape and climatic variables as well as host-species dynamics influence TBE infection risk in humans. The model was unable to explain some of the temporal variation, specifically the high counts in 2005 and 2006. Factors such as the exposure of humans to infected ticks and forest rodent population dynamics, for which we have no data, are likely to be explanatory factors. Such information is required to identify the determinants of TBE more reliably. Having records of TBE infection sites at a finer scale would also be necessary.

## Background

Tick-borne encephalitis (TBE) is the most important flavivirus infection of the central nervous system in Europe and Russia. The annual number of cases is estimated to be as high as 10,000 in Russia and about 3,000 in European countries [1-5]. Severe TBE infections caused by European virus strains typically take a biphasic course: After a short incubation period (usually 7-14 days, with extremes of 4-28 days), the first (viraemic) phase presents as an uncharacteristic influenza-like illness lasting 2-4 days (range 1-8 days) with fever, malaise, headache, myalgia, gastrointestinal symptoms, leukocytopenia, thrombocytopenia and elevated liver enzymes, and is often followed by a symptom-free interval of about one week (range 1-33 days). The second phase of TBE occurs in 20-30% of infected patients and is marked by four clinical features of different severity (meningitis, meningoencephalitis, meningoencephalomyelitis or meningoencephaloradiculitis) and the appearance of specific antibodies in the serum and cerebrospinal fluid. This is usually the time when patients with high fever and severe headache seek medical advice. The fatality rate in adult patients is less than 2%. However, severe courses of TBE infection with higher mortality and long-lasting sequelae, often affecting the patient's quality of life, are correlated with increased age [6-9].

**Figure 1:**
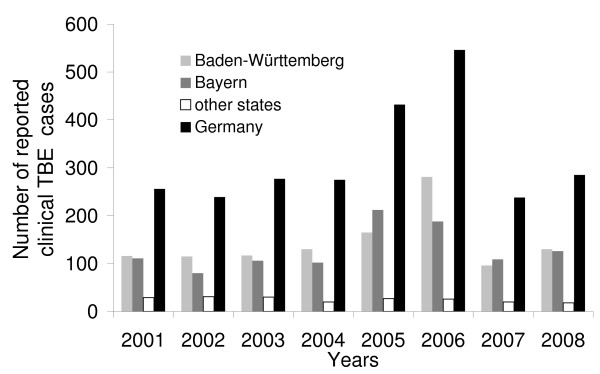
**Number of reported human TBE infections in Germany from 2001-2008**. Data source: Robert-Koch Institut: SurvStat, http://www3.rki.de/SurvStat. Accessed: 2/04/2009.

**Figure 2:**
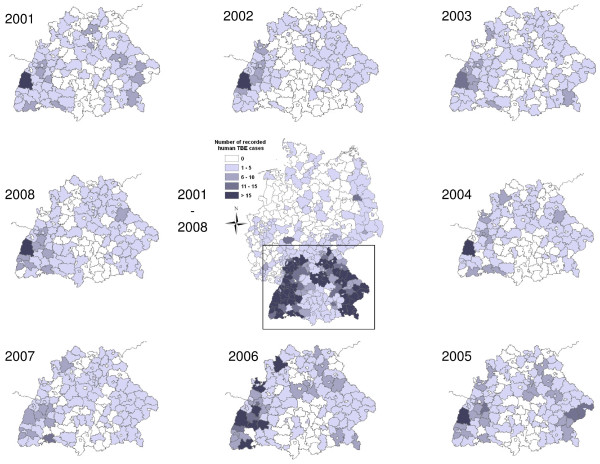
**Spatial and temporal variation in reported numbers of human TBE infections from 2001-2008**. The central map shows the distribution of TBE infection of entire Germany for the entire study period. Maps depicting the annual distribution of human TBE infections for the study area (States of Baden-Württemberg and Bavaria) are arranged clockwise around the central map. Data source: Robert-Koch Institut: SurvStat, http://www3.rki.de/SurvStat. Accessed: 02/04/2009.

As with most zoonotic diseases [10], TBE incidence in humans is characterised by considerable temporal (Figure 1) and spatial (Figure 2) heterogeneity [11]. The main determinant of infection risk is the density of infected ticks, i.e. the product of pathogen prevalence in the ticks and tick density [12]. *Ixodes ricinus*, the main vector of TBE virus (TBEV), has three distinct stages, larval, nymphal and adult ticks [13] whereas nymphs are responsible for the majority of tick bites in humans [14]. The *prevalence *of TBEV in (nymphal) ticks depends on a combination of factors; the basic reproduction number (R_0_) of TBEV, which crucially depends on the occurrence of non-viraemic virus transmission between ticks co-feeding, especially on forest rodents [15-17]. Furthermore, the persistence of TBEV depends on a threshold value between the density of competent (e.g. yellow-necked mice *Apodemus flavicollis*) and incompetent hosts (e.g. red deer *Cervus elaphus*) [18]. Possibly resulting from the species-specific reservoir competence, peaks in rodent populations in a given year are positively correlated with TBE incidence in humans in the succeeding year [19]. Co-feeding of larval and nymphal ticks on rodents critically depends on seasonal activity synchrony of these immature tick stages. Since Randolph et al.'s study [20], which related larval and nymphal synchrony to a certain temperature regime in autumn, subsequent research attention has been directed towards a specific temperature regime during spring time [11] causing the seasonal synchrony of immature ticks. Tick *density *in a given landscape is primarily determined by the availability of suitable forest habitat [21]. The findings of Allan et al. [22], relating to Lyme borreliosis risk, suggest that forest fragmentation might affect epidemiological risk. Tick density is further influenced by the abundance of host species such as roe (*Capreolus capreolus*) and red (*Cervus elaphus*) deer [23,24] which feed large numbers ticks in all stages of development [25,26]. Since estimates of wildlife population densities do not exist at the appropriate temporal and spatial resolution, we used hunting bag statistics as proxy for density. We also used hunting bag data of red fox (*Vulpes vulpes*), which was found to be positively correlated (with a time lag of one year) with TBE incidence in humans in Sweden [27]. The explanatory variables investigated in this paper include hunting bag data for red deer, roe deer and red fox, land cover, spring warming increase, and an index of forest fragmentation. Specifically we apply statistical tests, which take account of spatial correlation, to assess whether these factors are related to TBE incidence in humans.

## Methods

### Dependent variable

Annual symptomatic TBE infections in humans registered by the patients' place of permanent residence (county level) for the period 2001-2008 were obtained from the data base of the Robert Koch-Institut (SurvStat, http://www3.rki.de/SurvStat, 11/02/2009). We included the human population size for each county and year (provided by the federal statistical bureau) in our model. For a given county in a given year we modelled *p*, the proportion of reported clinical TBE cases *y *out of *n *inhabitants, assuming that TBE incidence in humans and infection risk are closely correlated.

### A model for the TBE-count data

Since our dependent variable consists of counts of successes (*y*) and failures (*n-y*) in *n *trials, we used a generalized linear model (GLM) with binomial error distribution, i.e. a logistic regression model [28]. The statistical package *R *[29] was used to estimate parameters of the model and to compute the test statistics. We model the probability that a person in a given county, and in a given year, is infected as a function of the explanatory variables.

### Explanatory variables

#### Forest type and forest cover

Information on forest type and forest cover for each county was obtained from the CORINE dataset with a spatial resolution of 100 meters [30]. We used the most recent available land cover information (year 2000) for all years, assuming that forest type and cover did not change substantially over this relatively short time period. For each county, we computed the proportion of coniferous, broad-leafed and mixed forest area.

#### Forest fragmentation

Given the relative coarse resolution of the CORINE dataset, we used the 'largest patch index' (largest forest patch/total forest area) to approximate the level of forest fragmentation for each county [31].

#### Spring warming

We extracted mean diurnal monthly interpolated temperatures from 30 arc s resolution climate surface maps based on the 1950-2000 period [32]. These data describe the spatial variation of temperatures which seems reasonable for our research question, given that temporal variation of temperatures is unlikely to explain the temporal variation of TBE incidence [33,34]. Data pre-processing included a validity check of the temperature data and a re-projection to the WGS84 cartographic system in GIS ArcView version 9 (ESRI, Redlands, CA, USA). Mean interpolated temperatures (in °C) were computed for each county, for the months of January, February, March and April. From these we computed the temperature increase in spring as the mean temperature increase between February and April corrected for the mean temperature of January [11].

#### Hunting bag data

We obtained annual hunting statistics of game species from wildlife authorities of both states (Baden-Württemberg and Bavaria) for the period 2000/2001-2007/2008. To deal with the unequal sizes of the 140 counties, we standardised the hunting bag counts by dividing them by area available for wildlife, the latter being approximated by the area covered by agriculture and forest. For Bavaria, hunting bag data on roe deer were not available for the period 2000/2001. Considering the minor temporal variation in roe deer hunting bags, we used the roe deer bag data of the period 2001/2002 also for the missing period. In Bavaria, roe deer hunting is based upon a three years management plan but hunting bags are approximately evenly realised among years. In Baden-Württemberg, roe deer hunting is based on yearly management plans. Management plans for carnivores are not in place.

#### Unexplained spatial and temporal variation

Spatial autocorrelation [35] was incorporated by using the proportion of TBE cases per inhabitant in the neighbouring counties as an explanatory variable in the model. To control for unexplained temporal variation, we included each year as fixed factor (with year 2001 as reference) in the model. Summary statistics of dependent and explanatory variables are provided in Table 1.

**Table 1:** Summary statistics of dependent and explanatory variables for a model explaining the spatio-temporal variation of tick-borne encephalitis in southern Germany from 2001-2008

Dependent variable	Mean	SD	Min.	Max.
Number of reported TBE infections in humans	1.95	3.26	0.00	39.00
County population size (in units of 10,000)	16.54	14.10	2.86	131.20
**Explanatory variables**				
***Proportion of forest cover/county***				
Broad-leafed forest	0.05	0.06	0	0.28
Coniferous forest	0.18	0.13	0	0.56
Mixed forest cover	0.09	0.07	0	0.36
***Forest fragmentation***				
Connectivity (largest forest patch/entire forest cover)	0.19	0.14	0	0.74
***Hunting bag in previous year per km^2 ^hunting area***				
Red deer *Cervus elaphus*	0.09	0.30	0	6.77
Roe deer *Capreolus capreolus*	4.46	1.30	0.76	10.10
Red fox *Vulpes vulpes*	2.17	0.91	0	17.86
***Climatic variables***				
Spring warming: Temperature increase from February-April (°C) corrected for mean January temperature	9.25	1.39	6.07	12.59
***Unexplained spatial variation***				
Total human TBE cases in all neighbouring counties per inhabitants of those counties	0.00013	0.00014	0	0.00091
***Unexplained temporal variation***				
Each year was entered as a fixed factor in the model	-/-	-/-	-/-	-/-

Note that the hunting bag of roe deer in the counties of Bavaria is the average annual offtake of three years.

### Modelling procedure

Before fitting a full model, we tested the effect of single covariates on the probability of TBE infections in humans after correcting for unexplained spatial and temporal variation (Table 2). We tested for multicollinearity between the explanatory variables (Table 3) by computing the condition number. For model checking the deviance residuals were computed and displayed using GRASS GIS [36].

**Table 2:** Relationships between single variables and the probability of tick-borne encephalitis infections in humans after correcting for spatial autocorrelation and unexplained temporal variation as tested by a logistic regression model

Parameter	Algebraic sign of coefficient estimate	Significance level
Proportion broad-leafed forest	-	0.10
Proportion coniferous forest	+	< 0.001
Proportion mixed forest	+	< 0.001
Connectivity	+	0.59
Red deer hunting bag	+	< 0.01
Roe deer hunting bag	+	< 0.001
Red fox hunting bag	-	0.24
Spring warming	-	< 0.001

**Table 3:** Correlation matrix (Pearson product-moment correlation coefficient) between variables potentially explaining the probability of tick-borne encephalitis infections in humans in southern Germany

Parameter	Proportion broad-leafed forest	Proportion coniferous forest	Proportion mixed forest	Largest forest patch index	Red deer hunting bag	Roe deer hunting bag	Red fox hunting bag
Proportion coniferous forest	-0.53						
Proportion mixed forest	0.33	-0.11					
Largest patch index	-0.13	0.17	-0.15				
Red deer hunting bag	-0.09	0.27	0.14	-0.06			
Roe deer hunting bag	-0.03	0.06	0.21	-0.34	-0.07		
Red fox hunting bag	0.20	-0.17	0.13	-0.17	-0.06	0.12	
Spring warming	-0.44	0.32	-0.33	-0.16	0.16	-0.02	-0.38

## Results

### Preliminary analyses

After correcting for unexplained spatial and temporal variation, five of the eight candidate variables were significantly (p < 0.05) related with TBE incidence in humans (Table 2). The candidate variables showed rather weak correlations, resulting in a condition number of 6.5, which indicates that multicollinearity is not of concern [37]. The final model was fitted using all candidate variables.

### Full model

**Figure 3:**
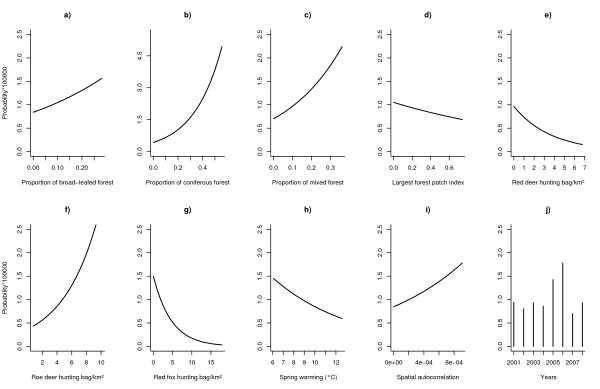
**Relative effects of significant variables of the final model explaining the variation of human TBE infections in southern Germany**. The effect of single variables on the probability of TBE incidence was estimated by predicting the full model on the entire range of the target variable. Non-target variables entered the model prediction with their mean value and using the year 2001 as reference. For a definition of the variables shown in a) - j), see Table 1.

The full model suggested that all candidate variables were significantly associated with TBE infection risk in humans (Table 4). The proportion of forest cover was positively correlated with probability of TBE infection; the effect of coniferous forest cover was stronger than that of broad-leafed and mixed forest cover (Figure 3a-c). When tested on its own, the proportion of broad-leafed forest cover was not significant (Table 2).

**Table 4:** Parameter estimates of variables derived from the full logistic regression model aiming at explaining the spatio-temporal variation in human tick-borne encephalitis infections in southern Germany from 2001-2008

Parameter	Estimate	Standard error	z-value
Intercept	-11.94***	0.32	-37.30
Proportion broad-leafed forest	2.21***	0.47	4.71
Proportion coniferous forest	4.38***	0.23	18.69
Proportion mixed forest	3.21***	0.32	10.04
Largest patch index	-0.58**	0.21	-2.76
Red deer hunting bag	-0.27*	0.11	-2.74
Roe deer hunting bag	0.21***	0.02	10.42
Red fox hunting bag	-0.21***	0.04	-4.97
Spring warming	-0.14***	0.02	-6.09
Spatial autocorrelation	817.75***	211.47	3.87
Year 2002	-0.16	0.10	-1.63
Year 2003	-0.02	0.10	-0.18
Year 2004	-0.09	0.09	-0.98
Year 2005	0.42***	0.08	4.92
Year 2006	0.64***	0.08	7.81
Year 2007	-0.30**	0.10	-2.93
Year 2008	-0.01	0.09	-0.13

(***P-value < 0.001; **P-value < 0.01; *P-value < 0.05).

TBE risk was significantly negatively associated with the forest fragmentation index. This suggests that, taking all other variables as fixed, TBE risk in humans would decline if forest cover were more continuous (Figure 3d). Admittedly, we did not detect a significant relationship between the forest fragmentation index and TBE risk if tested without other explanatory variables (Table 2).

Wildlife population densities (measured indirectly as described above) were significantly correlated with TBE risk in humans in the following year (Figure 3e-g). The correlation with roe deer density was positive but those with red fox and red deer were negative. Except in the case of red deer, all coefficients in the full model have the same sign as those in the corresponding restricted models. TBE risk was significantly correlated with temperature increase during the spring months (Figure 3h) both in the restricted and in the full model (Tables 2, 4). Unexpectedly, the correlation is negative.

There is strong evidence of spatial autocorrelation (Figure 3i) and, unfortunately, also of unexplained temporal heterogeneity. The incidence of TBE infections varied substantially from year to year (see Figure 1) peaking in 2005 and 2006. Of course we would have wished that the model could explain these peaks in terms of the explanatory variables considered here. As is evident in Table 4 we were unable to account for this temporal heterogeneity with the covariates available to us.

**Figure 4:**
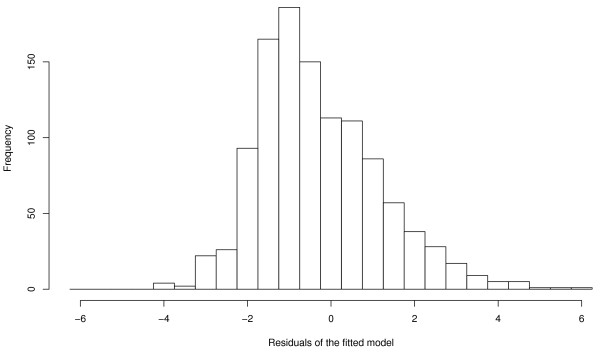
Histogram of the residuals of the final model explaining the variation of human TBE infections in southern Germany.

**Figure 5:**
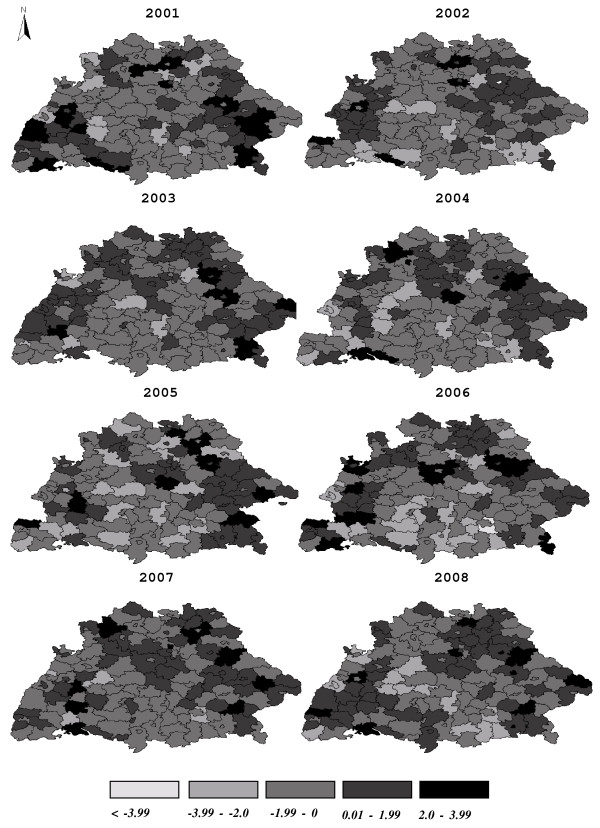
Spatial distribution of residuals derived from the negative binomial regression model on TBE infections in humans.

With the above limitations in mind, the model (Null deviance = 3580.0 on 1119 degrees of freedom; residual deviance = 2494.8 on 1101 degrees of freedom) explained a large fraction of the observed variance (Cox-Snell-R^2 ^= 0.96, Nagelkerke's R^2 ^= 0.65). A histogram of the deviance residuals of the model (Figure 4) indicates that the distribution of the residuals is somewhat skewed, which is due to the discrete nature of the response; about 40% of the observed counts are equal to zero, and almost 75% of them are two or less. The spatial distribution of the residuals (Figure 5) varied considerably from year to year. There was a slight tendency to underestimate the TBE incidence in counties with few reported cases (south central part of the study area) and to overestimate the incidence in counties with many reported cases (western, northern and eastern regions of the study area) (Figures 2 and 5).

## Discussion

The model explaining TBE incidence in southern Germany contains eight variables (three related to forest cover, one related to forest fragmentation, three related to wildlife species population dynamics and one related to climatic conditions during spring time) plus one variable each to account for unobserved spatial and temporal variation. With one exception (spring warming) the results are broadly in line with previous research, highlighting the range of factors influencing TBE transmission dynamics and thus infection potential in humans [33,34,38]. The fact that the factor "year" had to be included in the model constitutes a failure of the model. Other factors, especially those taking account of human activities, would need to be considered to explain the remaining and substantial temporal heterogeneity. The admittedly incomplete model does, however, consider the influence of several explanatory variables simultaneously, rather than one variable or a few variables.

The proposed model suggests that the proportion of coniferous forests had a stronger effect on TBE risk for humans compared to other forest types. Results from field studies provide equivocal results concerning the epidemiological risk [densities of (infected) ticks] according to forest type, but generally broad-leafed forest is considered as a suitable habitat for ticks. In North American forests, black-legged ticks (*Ixodes scapularis*) are more abundant in deciduous than in coniferous woodlands [39]. In Scotland, tick densities were highest in coniferous forests compared to deciduous woodland and pastures [40]. In Denmark, no differences in *Ixodes ricinus *tick densities between spruce (conifer) forests and non spruce forests were found [23]. It is, however, unlikely that forest type itself accounts directly for tick density patterns. Potentially, forest type indirectly affects tick densities by providing appropriate moisture and host species for ticks or by sustaining more TBE competent reservoir species relative to incompetent ones [18,23,33], or forest types are unevenly frequented by humans.

In addition to forest extent, we hypothesized that forest fragmentation would enhance TBE risk either by increasing density of infected ticks [22] or by enhancing contact rates between humans and ticks. The effect of forest fragmentation was unclear. Despite showing weak correlations with other variables (Table 3), which indicates that it is not confounded with other variables, the sign of the coefficient in the simple model was the opposite of that in the full model. Yet, removing this variable from the full model did not alter the signs of other coefficients.

Hunting bag densities of three game species were found to be significantly associated with TBE risk in humans. Since hunting bags are subject to management plans (deer) and/or influenced by hunting effort (red fox), these observations should be regarded cautiously [41]. Yet, "important ecological questions simply have to be addressed on the right scale - which often means an uncomfortable large scale - even if that means a certain degree of imprecision" [42]. There is growing empirical evidence that roe deer populations increase the epidemiological risk of ticks [23], Lyme disease risk [43] and TBE risk [[33], [44], this study]. Regarding the role of red deer in determining TBE risk, our results are inconclusive (changing signs simple model vs. full model) but do not necessarily contradict findings from Italy, where a non-significant effect was found between red deer and TBE incidence in humans [33].

The negative relationship found in this study between red fox hunting bags and human TBE cases in the succeeding year is in contrast to a study in TBE endemic areas in Sweden, where this relationship was found to be positive [27]. These contradictory findings will need to be clarified in future studies on the specific role of foxes in the epidemiology of TBEV.

Unfortunately, reliable data on forest rodent dynamics are not available at the appropriate temporal and spatial scale, and could thus not enter our model. Further attempts should be made to test the hypothesis that high rodent (especially *Apodemus flavicollis*) densities translate into high nymphal densities in the following year [45] and hence to elevate TBE incidence in humans [19]. Such a finding might be of use as an effective early warning system for public health.

Unexpectedly, the direction of the regression coefficient of the spring warming variable is not in line with the claimed importance of simultaneous activity of larval and nymphal ticks for TBE maintenance [11]. That theory suggests that fast warming in spring is required to allow nymphs (critical temperature ~7°C) and larvae (critical temperature ~10°C) to feed synchronously [16] and to transmit pathogens among the tick population [15]. However, it should be emphasised that spring warming has so far been used only to explain the distribution of TBE foci and not the variable incidence.

**Figure 6:**
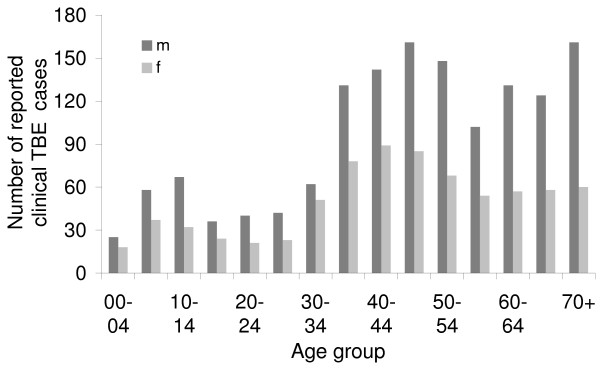
**Distribution of reported TBE cases in Baden-Württemberg and Bavaria by age group and sex (m = male, f = female)**. Data source: Robert-Koch Institut: SurvStat, http://www3.rki.de/SurvStat. Accessed: 02/04/2009.

As in most epidemiological studies, we based our analysis on the assumption that TBE incidence is strongly correlated with the epidemiological risk of TBE (i.e. the density of infected nymphs). Although the model provides a reasonable fit, some potentially important factors were not investigated due to lack of data. Included here are differing virulence of TBE, variable exposure of humans to ticks, differing immune responses, demography and TBE immunisation coverage. E.g. Kimmig et al. [46] found discrepancies in spatial distribution of clinical TBE cases and prevalence of TBE antibodies in sera of persons at risk. In addition, demographic analyses indicate that men above the age of 35 years are disproportional represented (Figure 6) among the persons with clinical TBE symptoms, which might be related to higher exposure risk to ticks and/or to differing immune responses [47]. Furthermore, locations associated with the counts available to us are the patients' place of permanent residence, and not the place of infection. Although it is likely that most infections occur in the infected person's home county, exceptions in this respect are clearly a potential source of bias. Due to data availability this analysis is based on a rather coarse scale [cf. 42]. Counties do not necessarily represent homogenous units with respect to environmental and ecological conditions. Generally forests, animal communities and interpolated temperatures are very heterogeneous within a given county. Thus any analysis based on values that have been averaged over variable conditions cannot be expected to yield precise results. Consequential we strongly recommend recording the suspected places of TBE infection as accurately as possible i.e. at the resolution of the forest patch.

## Conclusions

Our modelling approach focussed on ten biotic and abiotic factors possibly influencing the epidemiological risk of TBE. Except for the influence of spring warming, the results are in line with previously published findings. The presented approach might therefore be useful for predicting at least the potential direction, and approximate magnitude of TBE risk, as a function of ecological change (land cover change, wildlife population dynamics). To make further progress, a higher spatial resolution is needed in order to make more reliable interferences about the relationships between eco-environmental factors and TBE incidence. Moreover, neither this analysis nor the in-depth analyses of yearly temperature variability [33,34,48] could entirely explain the temporal dynamics of tick abundance and TBE in the past decade. This underlines previous notions that besides the epidemiological risk, the contact rate probability between infected ticks and humans is of crucial importance and should be incorporated in models for tick-borne diseases [11,43,48].

## Competing interests

The authors declare that they have no competing interests.

## Authors' contributions

CK designed the experiment, acquired the data, performed the statistical analysis, interpreted the results and wrote the manuscript. WZ designed the experiment, performed the statistical analysis, contributed to the interpretation of the results and the writing of the MS. PS performed the GIS analyses. FR acquired the data, contributed to the interpretation of the results and the writing of the MS. TV, PH and MN contributed to the interpretation of the results and the writing of the MS. All authors read and approved the final manuscript.
